# Brucellar Cervical Spondylodiscitis Complicated by Epidural Abscess and Neurobrucellosis

**DOI:** 10.1590/0037-8682-0556-2022

**Published:** 2023-02-20

**Authors:** Handan Alay, Mehmet Kürşat Karadağ, Bahar Yılmaz Çankaya

**Affiliations:** 1 Ataturk University, Faculty of Medicine, Department of Infectious Diseases and Clinical Microbiology, Erzurum, Turkey.; 2 Ataturk University, Faculty of Medicine, Department of Neurosurgery, Erzurum, Turkey.; 3 Ataturk University, Faculty of Medicine, Department of Radiology, Erzurum, Turkey.

**FIGURE 1:**
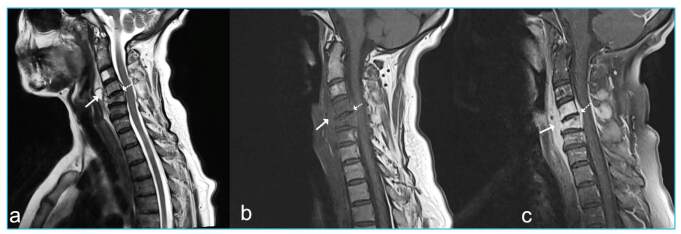
Pre-treatment cervical magnetic resonance images. **(a)** Non-contrast T1 AG without fat suppression. T1 AG images **(b)** Fat-suppressed contrast-enhanced; prevertebral abscess (arrow). **(c)** epidural abscess (dashed arrow).

**FIGURE 2:**
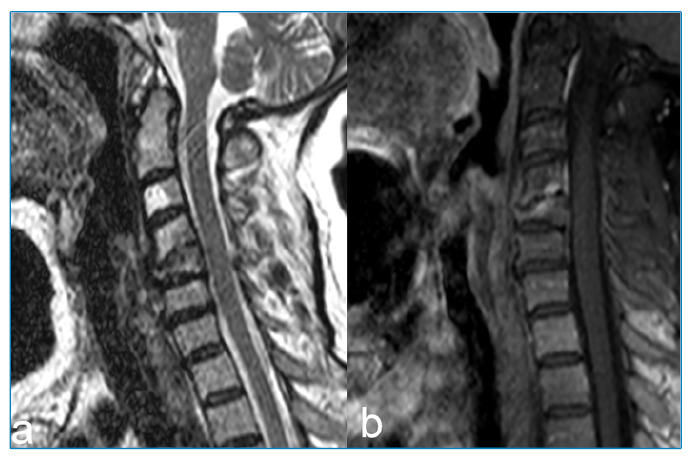
Post-treatment cervical magnetic resonance images. Almost complete regression can be seen in abscess formations in the prevertebral and anterior epidural distance on sagittal T2 AG **(a)** and fat-suppressed contrast-enhanced T1 AG **(b)** images

A 59-year-old woman diagnosis with brucellosis was admitted to our clinic with complaints of severe headache, neck pain, and pain and weakness in the left arm despite receiving treatment for 2 weeks. The patient participated in animal husbandry and had a history of making and consuming cheese from unpasteurized milk. Physical examination revealed a body temperature of 37 °C, painful neck vertebrae on local palpation, weakness of the left arm, and nuchal rigidity. Her white cell count was 6.07 × 10^3^/µL, C-reactive protein 57 mg/L, sedimentation rate 89 mm/h, Wright agglutination test 1/320, and *Brucella* IgM 0.40 and IgG 2.18 (cut-off value: 0.9-1.1). Cerebrospinal fluid (CSF) glucose was 64.6 mg/dL (blood glucose 120 mg/dL), microprotein 102.6 mg/dL, and lymphocyte count 4 cells/mm^3^. The patient was started on doxycycline 2 × 100 mg/day, rifampicin 1 × 600 mg/day, streptomycin 1 g/day, and ceftriaxone 2 × 2 g/day. No regression occurred in the patient’s shoulder and neck pain. The cervical magnetic resonance imaging results are shown in Figure 1. The CSF parameters were normal on day 20. After 6 months of treatment, almost complete regression was observed in abscess formation (Figure 2).

*Brucella* spondylodiscitis represents osteoarticular involvement of brucellosis and rarely involves the cervical region^1^. *Brucella*-related epidural abscesses and neurobrucellosis are rare complications with a poor prognosis^2^ ^,^ ^3^. Therefore, spondylodiscitis due to brucellosis, epidural abscess, and neurobrucellosis, which exhibits a wide range of clinical manifestations, should be considered in the differential diagnosis of individuals living in endemic areas.
